# Do health beliefs, personality traits, and interpersonal concerns predict TB prevention behavior among Japanese adults?

**DOI:** 10.1371/journal.pone.0211728

**Published:** 2019-02-22

**Authors:** Naomi Yoshitake, Mika Omori, Masumi Sugawara, Kiko Akishinonomiya, Sachiko Shimada

**Affiliations:** 1 Faculty of International Liberal Arts, Juntendo University, Tokyo, Japan; 2 Department of Psychology, Ochanomizu University, Tokyo, Japan; Universitá Cattolica del Sacro Cuore, ITALY

## Abstract

Despite public health measures and health-promotion efforts, the decline in tuberculosis (TB) morbidity in Japan has been slow, with a higher TB incidence rate relative to those observed in most developed countries. Because health behavior depends on multiple factors and is formulated within a social context, a theory-driven model would be necessary to increase TB prevention behavior. Based upon the Health Belief Model, this study examined the effects of health beliefs, personality traits, and social factors on TB prevention behavior among Japanese adults. A cross-sectional survey was carried out with a nationally representative sample (*N* = 911; 50.9% women; mean age 49.5, *SD* = 14.1). Path analyses gave empirical support for the hypothesized model, suggesting that TB prevention behaviors are influenced by not only perceived susceptibility to the illness but also social factors such as cues to action and one’s concern to benefit others. The findings have implications for research examining health communication tailored to individual differences in personality and interpersonal concern.

## Introduction

Tuberculosis (TB) is a major global health problem. Although TB appeared to have been almost eradicated in Japan, despite public health measures and health-promotion efforts, the decline in TB morbidity has been slow since 1975, and Japan has a mid-level TB burden, with 14.4 cases per 100,000 populations, which is much higher relative to those observed in most developed countries [1]. One of the factors hindering effective TB prevention behavior in Japan is the lack of efficient health communication strategies supported by theory-based research. Reviews have shown that interventions developed using theory exert more powerful effects relative to those without theoretical underpinnings [2], and there is an increasing emphasis on the identification and wide dissemination of evidence-based interventions [3]. A previous study applied the common-sense model [4] to TB prevention intention for female college students and community-dwelling women engaged in public health promotion activities [5]. However, few other studies have examined the applicability of health behavior theories to TB prevention behavior in the Japanese population.

### The health belief model and TB

The Health Belief Model (HBM) provides a framework to explain perceptions and attitudes that one has towards illness and the negative outcomes of certain actions. The theory assumes that people’s beliefs about the risk of contracting a disease or health problem, their perception of the effectiveness of proposed preventive behavior, and cues to action determine the likelihood that they will perform the behavior [6]. The model was expanded by Becker and Maiman’s [7] addition of modifying sociopsychological and demographic factors. In the field of TB prevention and control, the HBM was applied to study pulmonary tuberculosis examinations among indigenous nursing students in Taiwan [8], chest x-ray screening of Mexican migrant farm-workers [9], and TB care-seeking behavior among migrant workers in China [10]. A qualitative research applied the HBM and social cognitive theory to understand beliefs in migrant farm workers diagnosed with latent TB [11]. Rodriguez-Reimann et al. [12] tested the applicability of the HBM in explaining TB prevention behavior among Mexican Americans and found gender and acculturation differences in HBM constructs. Nonetheless, it is hard to find a study using HBM in TB research in Japan. In addition, few TB-related HBM research have vigorously tested Becker and Maiman’s hypotheses [7] including direct and indirect relationships among constructs and modifying role of sociodemographic and personality variables. There is a long-standing belief that individual differences in the experience of negative, unstable emotions (i.e., neuroticism) confer vulnerability to illness [13], whereas positive, stable emotions (i.e., agreeableness) are associated with better health-related quality of life [14] and future health [15]. However, previous studies have not evaluated the effects of personality traits on HBM variables and outcome behavior.

**Fig 1:**
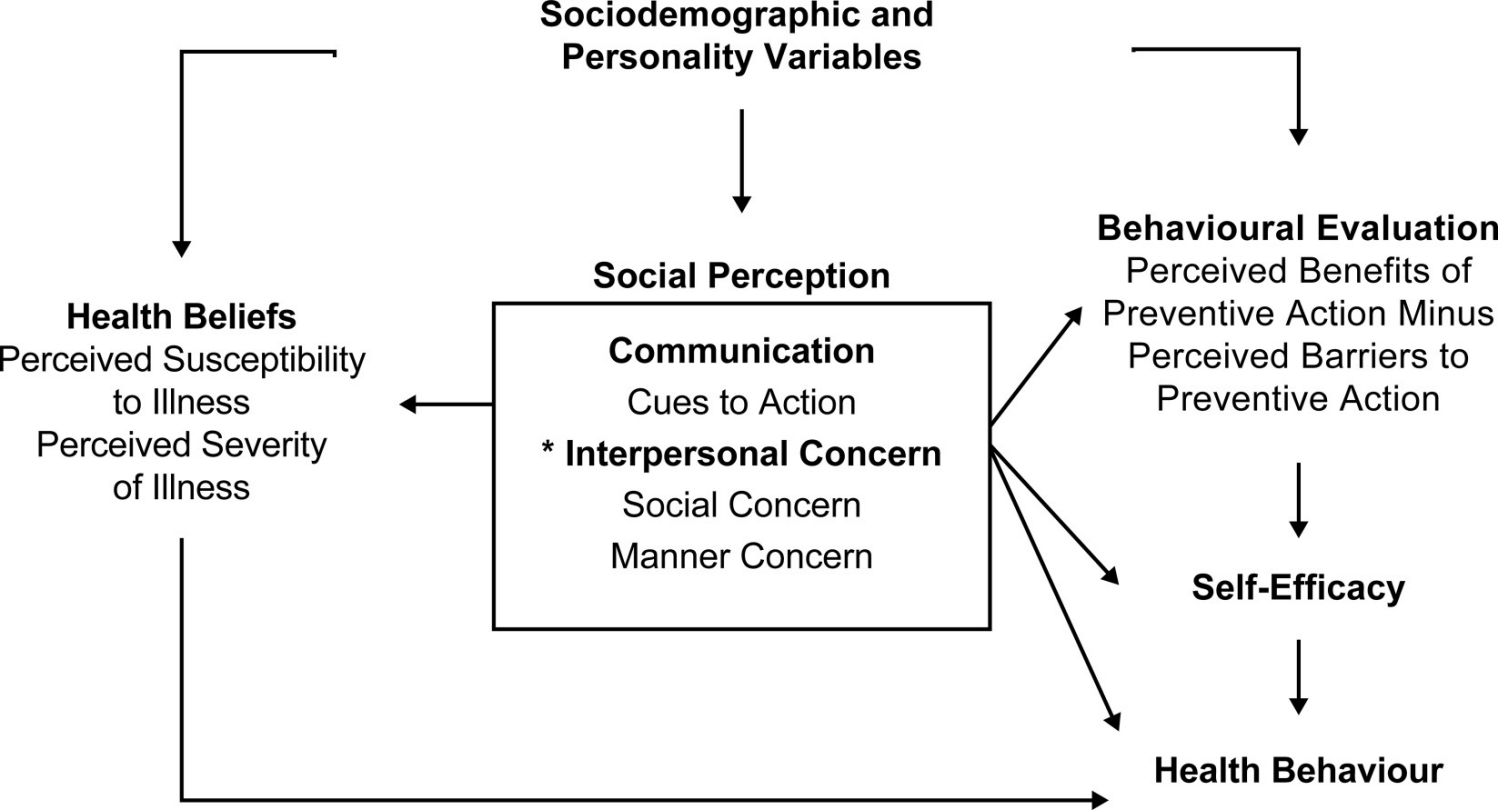
The hypothesized model based on the reconfigured health belief model developed by Mattson [16]. Asterisk (*) indicates newly introduced constructs for the study.

As an important development in HBM research, a study on a persuasive form of communication in counselling regarding HIV testing proposed a reconceptualization of the HBM [16]. Unlike the original HBM, in which the role of cues to action was considered cursory [15], the revised HBM model assumes that cues to action increase awareness of illness and influence other HBM variables, and it was therefore reconfigured as the core construct of the model (Fig 1). However, Mattson’s proposal [16] was examined using correlational analysis, and the reconfigured HBM structure has not been validated in TB prevention research thus far.

In non-Western cultures, illness perception appears to reflect social and moral factors [17]. Influenced by the collectivistic orientation of interdependence, cooperation, and mutual sharing [18], Japanese people are considered sensitive to social harmony and interpersonal etiquette and sometimes feel responsible for performing illness prevention behavior to ensure social regulation. Therefore, it is reasonable to assume that illness prevention behavior is directed not only by the health-related beliefs and attitudes [6], but also by one’s concern for society and the consequences of poor behavior. In particular, two types of interpersonal concern could affect prevention behavior: social concern and manner concern. Social concern refers to concern regarding social values and the consequences of one’s behavior for society, while manner concern refers to concern regarding poor behavior leading to social disapproval. The former is related to other-oriented attitudes that promote behavior intended to enhance the welfare of others, and the latter is related to self-oriented attitudes that control and inhibit behavior to fulfil social expectations [19]. These seemingly related but different types of interpersonal concern would exert unique effects on health beliefs and illness prevention behavior independent from cues to action in the HBM.

### The present study

Based on the reconfigured HBM framework, we structured a comprehensive model with health beliefs and perceptions, personality, and interpersonal concern, to explain TB prevention behavior among Japanese. The specific aims of the study were to: (a) empirically test reconfigured HBM for TB prevention behavior; (b) incorporate neuroticism and agreeableness into the HBM to examine individual differences in illness perception and prevention behavior; and (c) introduce social concern and manner concern as social perception components to the HBM. In accordance with health behavior models [20], we hypothesized that personality could moderate the extent to which health behavior change in response to health beliefs and interpersonal stimuli. Specifically, we postulated that individuals high in neuroticism have high anxiety level associating with negative health beliefs and unwillingness to engage in health behavior, whereas those high in agreeableness tend to practice frequent health behavior supported by their high social skills. Finally, although tentatively, because manner concern is focused on the behavioral aspect and social concern on the value aspect of morality, we expected that manner concern would exert a stronger effect on prevention behavior, relative to that exerted by social concern, in Japanese individuals.

## Materials and methods

The study was approved by the institutional review board at authors’ institution.

### Participants and procedure

We conducted a cross-sectional survey for Japanese adults aged between 20 and 70 years recruited via stratified random sampling. The country was geographically divided into 8 districts and each district was further classified into 3 strata based on the urban scale (i.e., metropolitan area with over 500,000 populations, urbanized area, and rural area). The number of sampling sites was calculated in accordance with estimated population sizes in each stratum in a district, yielding 77 sites in total nationwide. And from each sampling site, 30 individuals were selected randomly and invited to participate in a mail survey in 2015. Participation was voluntary, and the completion and return of the questionnaire implied the provision of informed consent. Because the survey was non-invasive and anonymous, the ethics committee approved the consent procedure. Participants received a JPY 500 book voucher for their participation. In total, 911 individuals participated in the study (response rate: 45.5%). The participants included 447 men (49.1%) and 464 women (50.9%), with a mean age of 49.5 (*SD* = 14.1) years. Approximately 7% of participants had completed junior high school, 41% were high school graduates, and 24% had completed junior college (23.7%) or university (24.6%). Approximately 45% worked full-time, 17.5% worked part-time, and 26% were unemployed. About 15% of participants had been in contact with TB patients (Table 1).

**Table 1:** Demographic characteristics of the participants (*n* = 911).

Characteristics	*n*	*%*
Gender (male)	447	49.1
Age		
<40	291	26.6
40–50	377	41.4
>60	292	32.1
Education		
Junior high school and lower	66	7.2
High school	372	40.8
College	216	23.7
University and above	251	27.6
Past history of TB	7	0.8
Past exposure to TB patients	139	15.3

### Measures

#### TB-specific HBM constructs

The HBM constructs were assessed via the adaptation of the Mexican American TB Health Belief Questionnaire [12]. The items assessed the following constructs: perceived susceptibility (four items; e.g. ‘My chance of contracting TB is high’), perceived severity (three items; e.g. ‘If I contract TB I might die’), perceived benefits (five items; e.g. ‘Having a chest X-ray will help me to detect TB early’), perceived barriers (five items; e.g. ‘Chest X-ray screening takes too much time’), and cues to action (seven items; e.g. ‘I am more likely to have a chest X-ray if my friends or family remind me’). Self-efficacy was assessed with seven items from the Self-Efficacy Scale for Mammography [21], which were modified to reflect participants’ confidence in their ability to undergo chest X-ray screening (e.g. ‘I can arrange transportation to have a chest X-ray’). Responses were provided using a 5-point scale ranging from 1 (*strongly disagree*) to 5 (*strongly agree*). Item scores were summed for each construct, and higher scores represented stronger beliefs. Because individuals’ evaluation of the efficacy of their behavior is assumed to reflect the action’s potential benefits weighed against the perceived cost involved in performing the proposed action [7], we calculated a composite score by subtracting the score for perceived barriers from that for perceived benefits, to produce a score for net benefits. Cronbach’s αs were .90 for cues to action, .86 for self-efficacy, .83 for susceptibility, .63 for perceived benefits, .64 for perceived barriers, and .57 for perceived severity. Although reliability coefficients for perceived benefits, barriers, and severity were low, given that TB-related health belief measures were adapted from a previous study to ensure consistency in TB research, and the removal of items did not improve the internal consistency estimates, these subscale scores were retained in further analyses.

#### Personality

Neuroticism and agreeableness were measured with Revised Neuroticism-Extraversion-Openness Personality Inventory [22]. Responses were provided using a 5-point scale ranging from 0 (*not at all true*) to 4 (*exactly true*). Mean trait scores were calculated according to the instrument manual [23]. Cronbach’s αs were .86 and .77 for neuroticism and agreeableness, respectively.

#### Interpersonal concern

The two types of interpersonal concern were assessed using 3-item scales widely used in social psychology research in Japan. The items assessing social concern [24] included ‘I sometimes think of the consequences that my actions have for society,’ ‘I sometimes think of how society is organized to function as a system,’ and ‘I sometimes think of the society I live in.’ The manner concern items [25] were ‘We should care for each other more in public places,’ ‘I am always concerned about bothering others,’ and ‘I will not pursue my goals if they might bother others.’ Participants’ responses were provided using a 5-point scale ranging from 1 (*not at all true*) to 5 (*very true)*. The sum of the subscale scores was calculated and higher scores indicated greater interpersonal concern. Cronbach’s αs were .77 for social concern and .62 for manner concern.

#### TB prevention behaviour

The outcome variable was measured using five items that assessed the frequency of the performance of specific types of behavior that aided TB prevention. The items were derived from TB brochures and public health websites in Japan [5], which were examined further by medical experts to determine whether they reflected the intended behavior. The items included ‘ventilating crowded rooms often,’ and ‘wearing surgical masks in crowds.’ Responses were provided using a 5-point scale ranging from 1 (*never*) to 5 (*always*), and item scores were summed to provide a total behavior score. Cronbach’s α was .78.

### Statistical analysis

Structural equation modelling was used to perform path analyses to examine the hypothesized relationships between variables, which were evaluated via maximum likelihood estimation using AMOS for Windows version 22.0. In specifying the model, demographic variables (i.e. sex, age, and educational level) and personality traits were allowed to correlate with each other and set to affect all constructs in the model. Nonsignificant results (*p* > .05) in the chi-square goodness of fit test indicated model adequacy. Comparative fit index (CFI) values greater than .90 and root mean square error of approximation (RMSEA) values below .08 indicate good fit [26].

## Results

The descriptive statistics and correlations are shown in Table 2. TB prevention behavior was associated with most of the HBM variables, except net benefits and neuroticism. Relative to the other variables, cues to action, social concern, and self-efficacy were more strongly related to TB prevention behavior. Age was positively related to cues to action, net benefits, self-efficacy, and prevention behavior and negatively related to manner concern. Higher educational levels were associated with lower levels of susceptibility and cues to action and higher levels of social concern, manner concern, net benefits, and TB self-efficacy.

**Table 2:** Descriptive statistics and correlations of the study variable.

	1	2	3	4	5	6	7	8	9	10	11	12
1. Susceptibility	–											
2. Severity	.34**	–										
3. Cues to action	.17**	.15**	–									
4. Social concern	.08*	.09*	.11**	–								
5. Manner concern	-.03	.13**	.04	.42**	–							
6. Net benefits	-.34**	-.07*	.09*	.05	.09**	–						
7. Self-efficacy	-.08*	.00	.19**	.09**	.07*	.38**	–					
8. Neuroticism	.17**	.19**	.01	.04	.22**	-.17**	-.12**	–				
9. Agreeableness	-.20**	-.08*	.06	.05	.20**	.27**	.13**	-.36**	–			
10. Prevention Behavior	.10**	.09*	.27**	.25**	.15**	.04	.22**	-.01	.10**	–		
11. Age	.03	-.04	.24**	.05	-.20**	.13**	.22**	-.28**	.14**	.17**	–	
12. Educational level	-.10**	-.03	-.09*	.19**	.10**	.13**	.07*	.03	-.03	-.01	-.22**	–
M	7.89	9.49	23.20	9.29	11.34	3.63	29.44	23.98	23.86	16.83	49.35	3.77
SD	2.90	2.35	6.48	2.28	1.72	4.65	5.18	7.38	5.38	4.33	14.12	1.02

*Note*: Education: 1 = primary school, 2 = middle school, 3 = high school, 4 = college, 5 = university, 6 = master’s degree, 7 = doctorate degree.

*p < .05

**p < .01.

**Fig 2:**
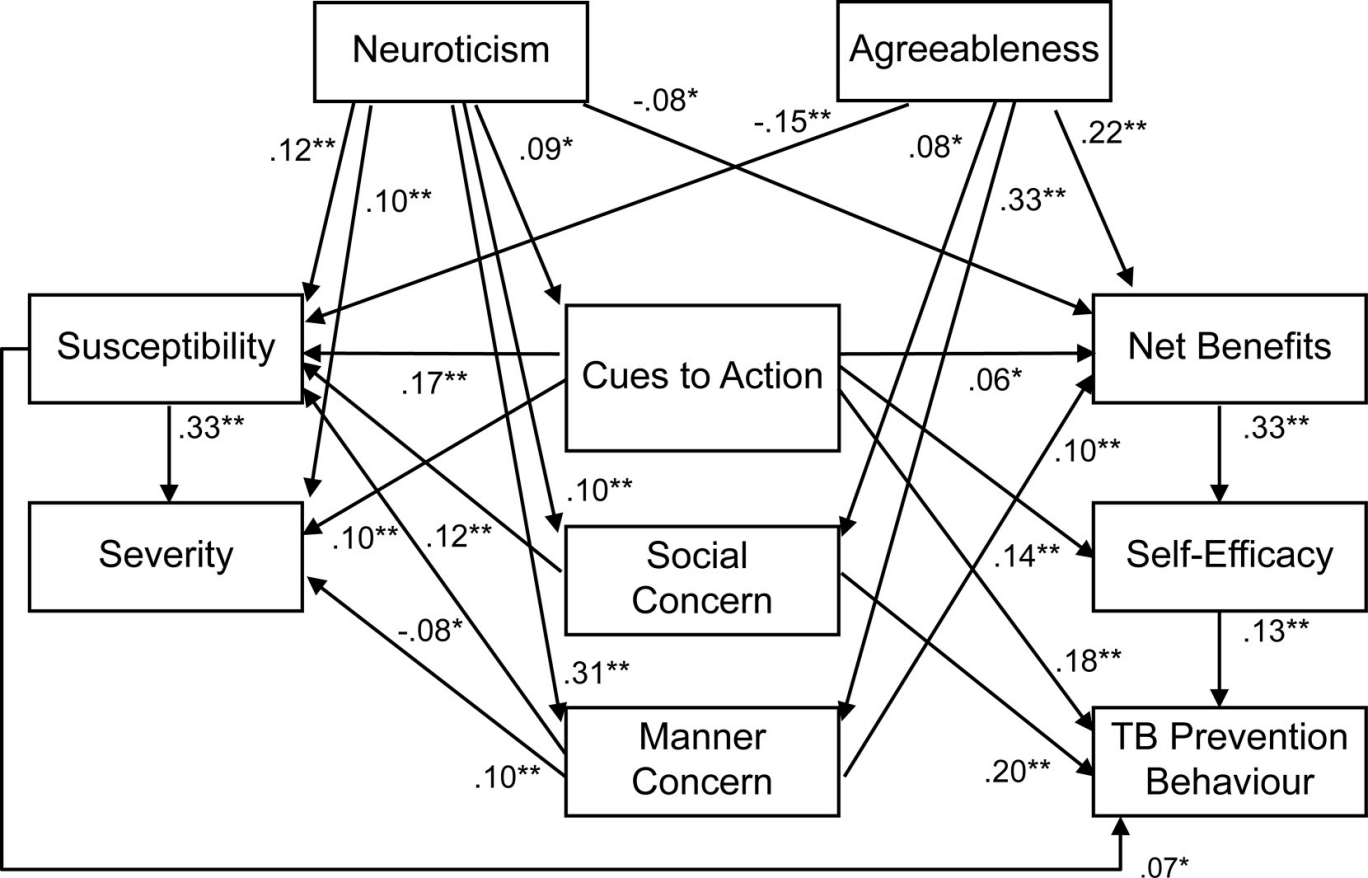
Estimation of the hypothesized health belief model for TB prevention behavior in Japanese adults. χ2 (*df*) = 6.64 (4), p = .16, CFI = 1.00, RMSEA = .03. **p < .01. *p < .05. Control paths by age, gender and educational level, covariance between error terms as well as non-significant paths are omitted.

The reconfigured HBM demonstrated a good fit to the data: χ^2^ = 4.05 (*df* = 4, *p* = .40), CFI = 1.00, RMSEA = .04, AIC = 104.05. The variables in the model explained 17% of the variance in TB prevention behavior. In addition, when the personality variables were added, the model demonstrated good fit: χ^2^ = 5.03 (*df* = 4, *p* = .29), CFI = 1.00, RMSEA = .02, AIC = 151.03, with an *R*^2^ value of .17. Furthermore, the hypothesized model that included social concern and manner concern demonstrated good fit: χ^2^ = 6.64 (*df* = 4, *p* = .16), CFI = 1.00, RMSEA = .03, AIC = 206.64, with an *R*^2^ value of .21. Although the final hypothesized model did not necessarily demonstrate a better fit relative to those of the previous models, it validated the addition of the two types of interpersonal concern as predictors in the traditional HBM. The final model is shown in Fig 2.

In accordance with the HBM hypotheses [6–7], susceptibility to TB was related to TB prevention behavior, and the perceived net benefits of prevention behavior were associated with TB screening self-efficacy, which in turn was related to TB prevention behavior. However, the severity of the health outcomes resulting from TB was not associated with prevention behavior. Furthermore, social concern was positively related to susceptibility and prevention behavior, while manner concern was associated with susceptibility, severity, and the perceived net benefits of prevention behavior. Neuroticism was positively related to susceptibility, severity, cues to action, social concern, and manner concern, and negatively related to net benefits, while agreeableness was positively related to net benefits, social concern, and manner concern and negatively related to susceptibility.

## Discussion

The present study examined the applicability of a comprehensive TB prevention behavior model that incorporated socio-cognitive and demographic variables based on the framework of reconfigured HBM [16]. This was the first study to assess the validity of the HBM in the general Japanese population. Elements identified as directly predicting people’s TB prevention behavior included a belief that they are susceptible to TB, concern about whether they exhibit socially desirable behavior, and confidence that they can undergo chest X-ray screening, as well as TB-related cues to action such as friends’ advice and newspaper spots. Significant relationships between personality and HBM components further highlighted that the addition of personality variables to the HBM was relevant.

In support of Mattson’s proposal [16], the analysis revealed that action cues were associated with all the other HBM variables, and that they were the focal point in the relationships between health beliefs and behavioral constructs. These results indicated three possible pathways via which communication cues affected TB prevention behavior: (a) it directly increased the likelihood of performing the behavior; (b) it indirectly affected the behavior by increasing perceived susceptibility to the illness; and (c) it affected both the perceived net benefits of the behavior and self-efficacy screening, which increased the likelihood of prevention behavior.

The results indicated that people high in neuroticism tended to respond to cues to action and interpersonal concern, perceive themselves as susceptible to TB, and consider the consequences of infection serious. Nevertheless, they believed that taking preventive action involved numerous difficulties. It is therefore plausible that prevention programs that address both accrued social costs aroused by avoiding preventive action and the ease of performing the behavior could result in prevention behavior in individuals with high neuroticism levels. In contrast, those high in agreeableness were aware of the interpersonal concern and benefits involved in taking preventive action, but perceived themselves as less vulnerable to TB relative to others. Therefore, strategies that challenge their health beliefs could result in more frequent prevention behavior.

Although exploratory, the results showed that, independent of the effect of cues to action, social concern and manner concern were associated with health beliefs and the behavioral component of the HBM, which provided support for health-related beliefs and behavior as social constructs. The results showed that TB prevention behavior was strongly correlated with social concern. This indicated that beliefs that emphasized harm to the community, rather than personal attitudes that inhibited behavior to avoid infection, triggered TB prevention behavior. A cross-national study showed that prosocial values increased participants’ intention to receive influenza vaccinations, thereby providing evidence that people were motivated to engage in prevention behavior to benefit others [27]. TB influences individuals’ lives and society, and people could engage in preventive action to increase their awareness of not only their own health risks, but also the risk of transmission to others.

The present study highlighted the importance of perceived vulnerability in the engagement in prevention behavior. Interestingly, perceived illness severity did not necessarily facilitate this behavior. These results support those of previous studies involving decision making regarding HIV testing [16]. Similarly, a review of the HBM [28] showed that perceived susceptibility predicted prevention behavior more effectively relative to sick-role behavior (i.e. actions taken to restore health after diagnosis), while perceived severity produced the lowest overall significance and was more strongly related to sick-role behavior relative to prevention behavior. These results suggest that risk perception should be personal (e.g. ‘you are vulnerable to the illness’), rather than threatening (e.g. ‘you could die from the illness’), to elicit prevention behavior. However, given the limited statistical power resulting from the low internal consistency of the severity subscale, future research is required to confirm the results.

The findings are likely to have been influenced by the Japanese TB control context. Therefore, additional research is required to examine the generalizability of the current findings. Further, the study could have benefited from the use of the full five-factor personality inventory to examine personality. Moreover, as the findings relied entirely on self-report data measured at one-time point, we could not infer causality in the relationships observed, and experimental and longitudinal studies are required to evaluate the causal processes between constructs.

## Conclusions

The study provided the first empirical evidence highlighting the important role that social factors play in explaining TB prevention behavior via the HBM. The findings also indicated that individuals with high neuroticism levels approached TB prevention behavior differently to those with high agreeableness levels. In addition, the effect of interpersonal concerns on HBM constructs suggested that framing prevention behavior as a social practice could be an effective means of health promotion, particularly for individuals who are sensitive to social harmony and the interests of others.
